# PsVPS1, a Dynamin-Related Protein, Is Involved in Cyst Germination and Soybean Infection of *Phytophthora sojae*

**DOI:** 10.1371/journal.pone.0058623

**Published:** 2013-03-14

**Authors:** Delong Li, Zhijian Zhao, Yidan Huang, Zhaojun Lu, Meng Yao, Yujuan Hao, Chunhua Zhai, Yuanchao Wang

**Affiliations:** Key Laboratory of Monitoring and Management of Crop Diseases and Pest Insects, Ministry of Agriculture, and Department of Plant Pathology, College of Plant Protection, Nanjing Agricultural University, Nanjing, China; Virginia Tech, United States of America

## Abstract

Plant pathogens secrete effector proteins to suppress plant immunity. However, the mechanism by which oomycete pathogens deliver effector proteins during plant infection remains unknown. In this report, we characterized a *Phytophthora sojae* vps1 gene. This gene encodes a homolog of the *Saccharomyces cerevisiae* vacuolar protein sorting gene vps1 that mediates budding of clathrin-coated vesicles from the late Golgi, which are diverted from the general secretory pathway to the vacuole. *PsVPS1*-silenced mutants were generated using polyethylene glycol-mediated protoplast stable transformation and were viable but had reduced extracellular protein activity. The *PsVPS1*-silenced mutants showed impaired hyphal growth, and the shapes of the vacuoles were highly fragmented. Silencing of *PsVPS1* affected cyst germination as well as the polarized growth of germinated cysts. Silenced mutants showed impaired invasion of susceptible soybean plants regardless of wounding. These results suggest that *PsVPS1* is involved in vacuole morphology and cyst development. Moreover, it is essential for the virulence of *P. sojae* and extracellular protein secretion.

## Introduction

Plant pathogenic oomycetes such as *Phytophthora* have unique physiological characteristics and devastating effects on crops and natural ecosystems [1]. To date, over 100 *Phytophthora* species have been described [2], including *P. infestans*, which causes potato late blight, the disease responsible for the great Irish potato famine of the 1840s [3]. Another representative is *P. sojae*, which causes soybean root and stem rot and approximately $1–2 billion of damage globally each year [4], [5]. The initial step in plant infection is the successful penetration of the plant surface. This process involve specialized infection structures called appressoria, which can release enzymes that degrade the walls of plant cells. Once the infectious hyphae enter the plant cell, they form haustoria. Pathogen invasion is often accompanied by host recognition, usually triggering a defense response. The defense response can be countered by pathogens through several mechanisms. For example, the suppression of specific signal transduction or gene expression processes in plant cells can protect the pathogenic enzymes. In addition, the pathogen can secrete a large set of effector molecules that interact with the host and establish compatibility.

Expression of the *Magnaporthe grisea* effector Avr-Pita, as well as the *Melampsora lini* effectors AvrL567 and AvrM, in the cytoplasm of resistant plants has been shown to trigger cell death [6], [7]. Pep1 and Pit2, which are secreted effector proteins of *Ustilago maydis*, are important for the successful invasion of plant cells [8],[9]. The Avr3a effectors of *P. infestans*, as well as Avr1b and Avh331 from *P. sojae*, are known to suppress programmed cell death and contribute to virulence [10]. Effectors are small secreted proteins lacking conserved functional domains in fungi. However, 10 oomycete Avr effector proteins were all found to share an N-terminal RxLR-dEER motif, which mediates translocation of these proteins into host cells [10]. Many play critical roles in plant–pathogen interactions. Recently, a total of 169 *P. sojae* effector proteins have been identified using an *Agrobacterium tumefaciens*–mediated transient expression assay in *Nicotiana benthamiana* [11]. Although secreted effectors are known to play an important role in pathogenesis, little is known regarding the sorting processes and secretion pathways by which these proteins reach their final destination. It is known that secreted proteins are produced in the endoplasmic reticulum and are secreted through Golgi-derived vesicles [12]–[16]. MgAPT2 from *M. grisea*, belonging to the type IV Drs2 family of aminophospholipid translocases (APTs), are required for efficient Golgi function and are involved in the exocytosis of virulence-associated proteins [17]. During the sorting process, the protein sorting apparatus can recognize targeting signals on soluble lysosomal proteins and guide them to the lysosome.

In *S. cerevisiae* mutants, carboxypeptidase Y (CPY) is sorted differentially through a pre-vacuolar compartment (PVC) prior to exocytosis. Vacuolar protein sorting (VPS) mutants have been identified, which secrete proenzyme forms of soluble vacuolar hydrolases to the cell surface and exhibited disparity in CPY sorting defects [18]. To examine the secretion of extracellular proteins in *P. sojae*, several homologs of *S. cerevisiae* pre-vacuolar secretory genes were identified, including the late-Golgi vacuolar protein sorting gene vps1, which is a dynamin implicated in the formation of vesicles destined to the endosomes from the trans-Golgi network. VPS1 was originally identified in a screen for *S. cerevisiae* mutants that affect vacuole biogenesis [19]. In the filamentous fungi *Aspergillus nidulans*, vpsA (a homologue of *S. cerevisiae* vps1) also played a role in vacuolar biogenesis [20]. However, in *Candida albicans*, it contributed to virulence-related phenotypes [21]. Vps1 is known to function in several membrane trafficking pathways including Golgi and vacuolar protein sorting. Vps1 regulates vacuole fission, peroxisomal fission, fusion, and tubulation in fission yeast [22]–[24].

In this study, PsVPS1, a homolog of the yeast dynamin-related protein VPS1, was identified in *P. sojae* and contained a conserved N-terminal GTPase activity domain and a GTPase effector domain (GED) at the C-terminus [25], [26]. Our results indicate that *PsVPS1* is essential for virulence and plays a role in the secretion of extracellular protein in *P. sojae*. In addition, silencing of *PsVPS1* impaired vacuolar biogenesis and cyst polarity growth. Our results suggest that *PsVPS1* plays a different role in the development and pathogenesis of *P. sojae*.

## Materials and Methods

### Gene Identification and Sequence Analysis

DNA and protein sequence predictions were obtained for the following species: *P. sojae*, *P. ramorum*, *and P. capsici* from the Joint Genome Institute (JGI) (http://www. jgi. doe. gov/); *P. infestans*, *Phaeodactylum tricornutum*, *Mus musculus*, *Magnaporthe oryzae*, *Homo sapiens*, and *Thalassiosira pseudonana* from the National Center for Biotechnology Information (NCBI) (http://www.ncbi.nlm.nih.gov/); *Saccharomyces cerevisiae* from the yeast genome (http://www.yeastgenome.org/); and *Arabidopsis thaliana* and *Oryza sativa* from the Broad Institute (http://www.broadinstitute.org). BLAST searches were performed against the above-mentioned genome sequence databases [27]. Protein sequences of fungal VPS1s were used for the TBLASTN searches of the *P. sojae* genome. All approaches generated a similar set of genes. The candidate sequences were determined through manual revision based on sequence conservation and were confirmed by submitting them to Prosite (http://www. expasy. org/prosite) to identify conserved function-discriminating residues.

Conserved domain searches were performed using the NCBI CD search program (http://www. ncbi. nlm. nih. gov/Structure/cdd/wrpsb. cgi) [28]. Alignments were generated using Clustal X [29], [30], and phylogenetic dendrograms were constructed using MEGA 4.0 [31] with the neighbor-joining algorithms and 1000 bootstrap replications.

### *Phytophthora sojae* Strains and Culture Conditions

In this study, *P. sojae* genome sequencing strain P6497 (Race 2), provided by Prof. Brett Tyler (Virginia Bioinformatics Institute, Blacksburg, VA), was used as the wild-type strain. The wild-type and silenced mutants were routinely grown on 10% V8 medium at 25°C in the dark, as described previously [32]. For growth assays, the recipient strain P6497 and the silenced mutants were sub-cultured twice and then cultured on 10% V8 juice agar medium. We obtained asexual samples including hyphae, zoospores, and germinated cysts as described previously [33]. The oospores were obtained on 10% V8 juice agar medium at 25°C in the dark for 10 days. For the extracellular laccase assay, wild-type and silenced mutants were cultured on lima bean agar medium containing 0.4 mM ABTS (2, 2′-azino-di-3-ethylbenzathiazoline-6-sulfonate), which used as a substrate with hydrogen peroxide for a peroxidase enzyme [34].

To examine the germination of zoospores, 500 µL of zoospore suspension was incubated in 5% V8 liquid medium in 1.5 ml tubes and vortexed for 90 s to induce encystment. The tubes were incubated at 25°C in the dark for 12 h. At least 100 cysts were examined for each treatment, and all treatments were performed in triplicate.

### Plasmid Construction and *P. sojae* Transformation

Full-length *PsVPS1* ORF was amplified using PrimeStar polymerase (TaKaRa) from cDNA with oligonucleotides primers *PsVPS1*F (5′-ttatcgatatggaccagctgatcccc-3′) and *PsVPS1*R (5′-ttcgtacgtcacttaaacgtattgaag-3′). The amplification fragment digested with *ClaI* and *BsiwI* was ligated in sense orientation into the pTOR vector and was sequenced to confirm the open reading frame and produce the plasmid pTOR-*PsVPS1*.

We transformed *P. sojae* using a polyethylene glycol-mediated protoplast transformation strategy with the target plasmids [35], including the sense construct of pTOR-*PsVPS1*. Putative *P. sojae PsVPS1* mutants were screened, and RT-PCR was performed on RNA extracted from each line using the oligonucleotides VPS1QF (5′-gtcgtcaagcttcctcaggt-3′) and VPS1QR (5′-cgagctcgttctgaatattc-3′).

### RNA Manipulation of *P. sojae*

Total RNA was isolated using the OMEGA (R 6834-1,U.S.A.)RNA extraction kit following the manufacturer’s protocol. To investigate the efficiency of *PsVPS1* gene silencing in the putative transformants, quantitative RT-PCR was performed. First strand cDNA was synthesized from 1 to 5 ug of total RNA by oligo(dT) priming using an M-MLV reverse transcriptase kit (Invitrogen, U.S.A.) following the manufacturer’s protocol. For gene expression analysis, SYBR green qRT-PCR assays were performed on the ABI 7500 real-time PCR system (Applied Biosystems, Foster City, CA, U.S.A.) according to the manufacturer’s instructions. A 20-µl reaction volume contained 2 µl of reverse transcription product, 10 µl of SYBR premix Ex Taq (2×), 0.4 µl of ROX reference dye (50×, SYBR Prime Script RT-PCR kit; TaKaRa), and 0.4 µl of each primer (10 µM). The actA gene from *P. sojae* was used as a constitutively expressed endogenous control, and the expression of each gene was determined relative to actA using the ΔΔCt method. qRT-PCR experiments were repeated in triplicate with independent RNA isolations.

### Staining

To examine vacuole morphology, strains were incubated in 10% V8 juice medium at 25°C in the dark for 2 days. The hyphae were then stained by CMAC (1 µM, c2210, Invitrogen) for 30 min and washed twice with PBS. The dyed hypha were mounted on microscope slides and examined under inverted fluorescence microscope (Olympus IX71, Japan) with a 40× objective, using ultraviolet excitation cube (330–385 nm excitation, >420 nm emission).

To examine cyst germination morphology, cellulose were stained with Congo Red (Sigma-Aldrich, U.S.A; 0.5% in water) for 5–10 minutes and washed in distilled water [36]. Samples were then mounted on microscope slides and observed using an Olympus microscope. Calcofluor White (CFW) (Sigma-Aldrich, St. Louis, MO, U.S.A.) staining was also used to stain polysaccharide, including cellulose and chitin, as described previously [37]. Photographs were taken using an Olympus microscope.

### Virulence Test

For plant inoculation, P6497 and *PsVPS1*-silenced mutants were incubated in 10% V8 broth in the dark for 2 days. Zoospores were then induced by washing with sterilized distilled water (SDW). The soybean cultivar Hefeng 47, which is compatible with *P. sojae* strain P6497, was planted in plastic pots containing vermiculite in the dark for 4 days in the greenhouse. Wounded or unwounded hypocotyl of etiolated seedlings was inoculated with 100 zoospores, and then etiolated seedlings were maintained in 80% humidity and darkness at 25°C. Two days post-inoculation (dpi), the pathogenicity results were investigated and photographs were taken.

Detached soybean leaves of Hefeng 47 were placed in Petri dishes. Each wounded or unwounded leaflet was inoculated with 100 zoospores. The leaves were incubated in a growth chamber at 25°C under 80% humidity in the dark for 48 h. Pictures of the lesions were taken at 2 dpi. The same leaflet was stained by Trypan Blue (Sangon Biotech, Shanghai), as described previously [38], followed by destaining with alcohol, after which the samples were photographed.

Trypan Blue staining was performed on the hypocotyl of etiolated seedlings infected with 100 zoospores at different time points. After 12, 24, and 48 hours post-inoculation (hpi), the infected cells of the epidermis were ripped and soaked in Trypan Blue, followed by destaining in chloral hydrate. The dyed infected epidermis cells were examined using light microscopy. All assays were repeated at least three times.

## Results

### *PsVPS1* is a Dynamin-related Protein and is Evolutionarily Conserved in Eukaryotes

**Figure 1:**
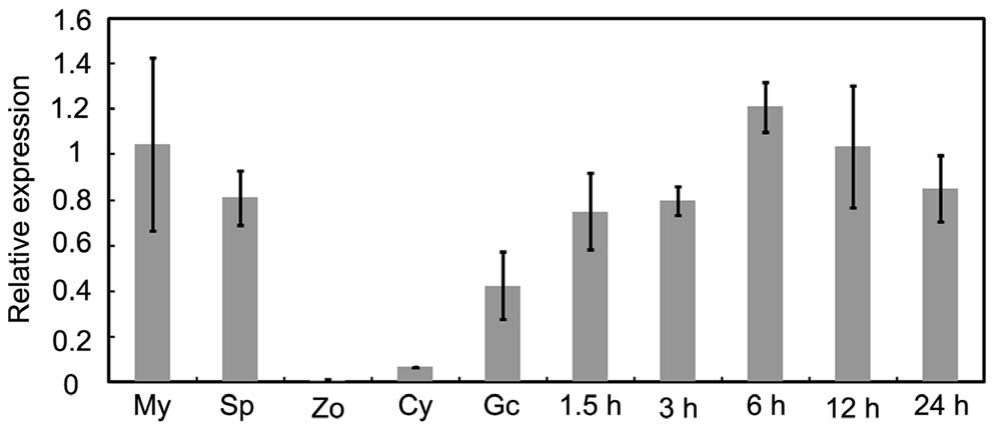
*PsVPS1* expression during different developmental and infection stages. RT-PCR was used to examine *PsVPS1* expression levels from vegetative hyphae (My), sporulating hyphae (Sp), zoospores (Zo), cysts (Cy), germinating cysts (Gc), and infected susceptible soybean cultivar samples at 1.5, 3, 6, 12 and 24 hpi.

The expression patterns of all *P. sojae* dynamin-related genes were obtained from the *P. sojae* transcriptional database [39]. We identified a gene encoding a dynamin-related protein with similarity to the *S. cerevisiae* VPS1 gene, which we named PsVPS1 (Protein ID:109490). This gene was 2127 bp in length, producing a 708 amino acid protein which was highly expressed in hyphae. On the basis of the expression patterns, real-time quantitative PCR was employed to analyze the expression of *PsVPS1* during distinct asexual developmental and infection stages. We found that *PsVPS1* was up-regulated during five *P. sojae* infection stages compared to zoospore and cyst stages, suggesting that it plays an important role during infection (Fig. 1).

**Figure 2:**
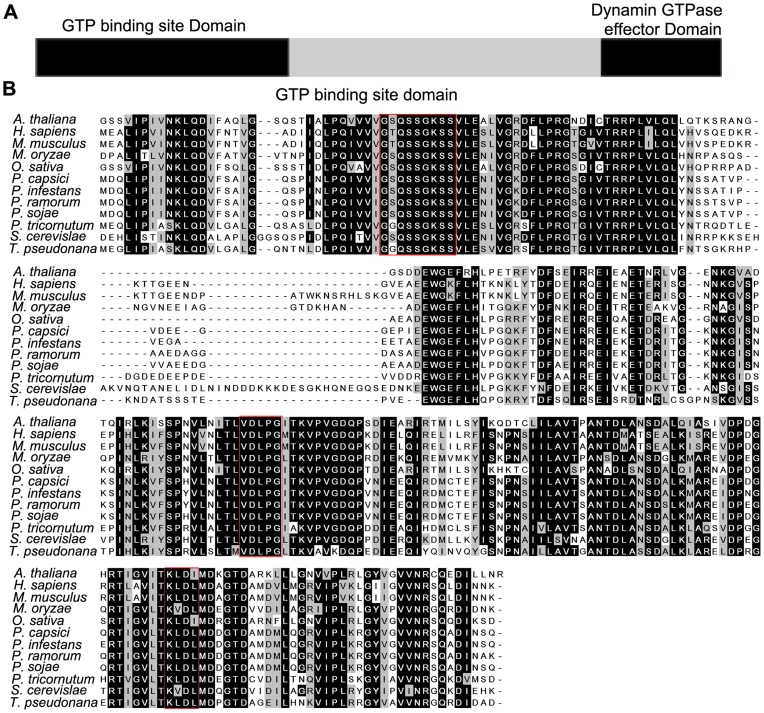
Functional domain comparisons of VPS1 in different organisms. **A.** Functional domain comparison and phylogenetic tree construction of vps1 in different organisms. Physical map of PsVPS1 showing conserved functional domains and the GTP binding site domain spanning amino acids 1 to 254. The dynamin GTPase effector domain spans amino acids 610 to 701. **B.** Alignment of VPS1 from *P. sojae* with different organisms was performed using the Clustal W program. Identical amino acid residues are highlighted against black background shading, similar amino acid residues are shaded with a light gray background, and distant similar amino acids are not shaded. The consensus sequences for GTP-binding are boxed.

The *S. cerevisiae* genome encodes three DRPs (dynamin-related proteins), Vps1, Dnm1, and Mgm1, all of which contain the N-terminal GTP site binding domain, a middle domain, and a GTPase effector domain (GED) at the C-terminus [40]. Unlike other classical dynamin-like proteins, the PH domain is absent, but the C-terminal proline-rich domain was found in VPS1. In *P. sojae*, we identified a conserved dynamin-related protein PsVPS1, similar to VPS1 of *S. cerevisiae* on AA level, which contained three conserved domains. The protein sequences of VPS1 were highly conserved in eukaryotic organisms, as shown in Fig. 2A. Alignment of the amino acid sequences of VPS1 from different species revealed that *PsVPS1* was highly conserved with conventional dynamins within the GTP-binding motifs (Fig. 2B). The vps1 of *P. sojae* was 45% identical to VPS1 of *S. cerevisiae*, 58% identical to *P. tricornutum*, 47% identical to *A. thaliana*, and 96% identical to *P. infestans*. Comparison of the phylogenetic tree based on the full protein sequence to vps1 from different organisms revealed that the *PsVPS1* sequence was distant from animal sequences and fungi, including *Magnaporthe oryzae* (XP_364672) and *Saccharomyces cerevisiae* (NP_012926) (Fig. S1).

### *PsVPS1*-silenced Mutants Showed Reduced Vegetative Growth and Altered Vacuole Morphology

**Figure 3:**
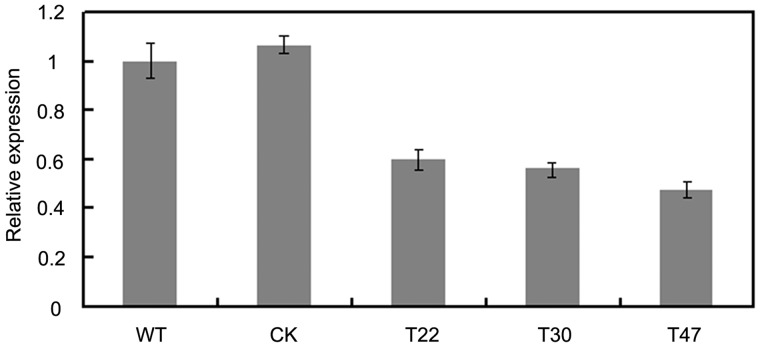
Expression analysis of *PsVPS1*-silenced mutants. qRT-PCR evaluation of *PsVPS1* gene (gray box) expression levels using hyphal RNA from recipient strain 6497, the control transformant (CK), and *PsVPS1*-silenced mutants (T22, T30, and T47). Expression was calibrated against the wild-type strain 6497 (value of 1). The qRT-PCR experiment was repeated three times with independent RNA isolations.

To generate *P. sojae* transformants lacking *PsVPS1*, a gene-silencing strategy was used as described previously [33], [41]. *Phytophthora sojae* strain 6497 was transformed with a plasmid containing the *PsVPS1* coded region in sense orientation under control of the HAM34 promoter and terminator, and carrying the Geneticin resistance gene NPT under control of the HSP70 promoter and terminator [10], [33], [42], [43]. In total, more than 120 independent transformants that could grow in the selection medium with 50 µg/µL Geneticin (Shanghai Sangon,BS723) were selected. Subsequently, the putative mutants were tested by real-time qPCR. The expression of *PsVPS1* was significantly reduced in the three mutants (T22, T30, and T47), and the silencing efficiencies were 40%, 44%, and 53%, respectively, compared to the wild type (P6497) (Fig. 3). The silencing efficiency was low because the expression of PsVPS1 during the mycelium stage was high that means vps1 is an essential gene for pathogen. Thus, we obtained three partially silenced mutants that could be used to analyze the function of PsVPS1 in future studies.

**Figure 4:**
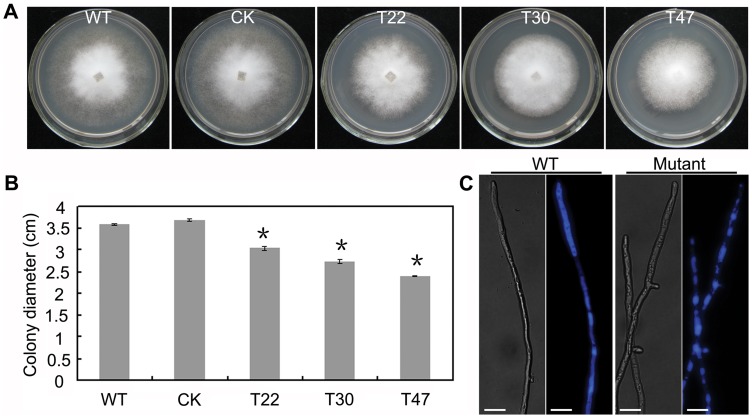
*PsVPS1*-silenced mutants showed reduced vegetative growth and vacuole morphology. **A.** The transformants displayed retarded vegetative growth on 10% V8 medium. Pictures were taken 4 days after incubation. **B.** Colony diameter after 4 days of growth (p<0.01). **C.** Vps1 is involved in vacuole size. Transformants had smaller vacuoles than did wild-type strains. Wild-type and transformant cells were stained by CMAC. Hyphal vacuoles of wild-type and transformant strains after 2 days of growth in 10% V8 liquid medium were stained by CMAC. The dyed hyphae were examined using the Olympus ultraviolet excitation cube (330–385 nm excitation, >420 nm emission). Bar indicates 50 µm.

To analyze the role of *PsVPS1* on mycelium growth in *P. sojae*, the three silenced mutants and wild-type 6497 were grown on 10% V8 medium at 25°C. To compare their growth ratio after 4 days, the colony diameters were measured. After 4 days on 10% V8 media, the silenced mutants displayed reduced growth compared to P6497 with colony diameters of 3.6 cm (P6497), 3.7 cm (CK), 3.0 cm (T22), 2.7 cm (T30), and 2.3 cm (T47), respectively (Fig. 4A and B). Mutant growth also significantly decreased (*P*<0.01), but the morphology of silenced mutant colonies was not affected.

Vps1 plays a role in maintaining vacuole function in budding and fission yeast, but also plays a role in the vacuole of filamentous fungi [19], [20]. To investigate the role of *PsVPS1* in the vacuole of *P. sojae*, CMAC (7-amino-4-chloromethylcoumarin) was used to specifically isolate the vacuoles (Fig. 4C). We found that silenced mutants contained some fragmented large vacuoles surrounded by smaller vacuolar compartments. In contrast, in the wild-type strain 6497, single large vacuoles were observed, with diameters similar to the mycelium. This indicated that *PsVPS1* was necessary for vacuole morphology in *P. sojae*.

### Germinated Cyst of *PsVPS1* Showed Impaired Polarized Growth

**Figure 5:**
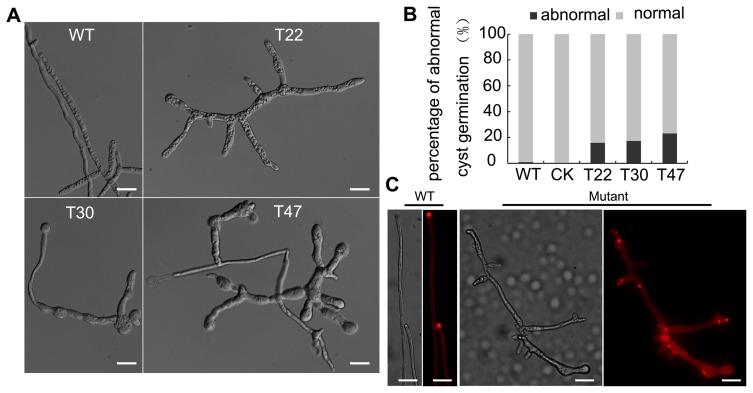
*PsVPS1* was required for normal zoospore germination. **A.** Zoospores of the transformant displayed abnormal morphology. Zoospores from the wild-type and *Psvps1*-silenced mutants (T22, T30, and T47) were encysted by vortexing for 90 s, and cysts were incubated in clarified 5% V8 liquid medium at 25°C for 12 h, after which samples were photographed. **B.** Numbers of abnormal germinated cysts were counted at 12 h under the microscope. The ratio of the number of abnormal germinated cysts to the total number of cysts (germinated and ungerminated) was calculated. **C.** Silencing of *PsVPS1* altered the distribution of β-1,4 polysaccharides in the cell wall. In silenced mutants, stained patches were detected along the hyphae and hyphal tips, indicative of abnormal polysaccharide accumulation. Bar indicates 20 µm.

Because *P. sojae* zoospores are the primary sources of infection, asexual development is critical to sustain the polycyclic disease cycle. Zoospores can shed flagellum and form cysts, then cysts germinate to produce infectious hypha or to form appressoria that responds to physical signals to penetrate the host. The germinated cyst is important for polar growth of hyphae and is the first step of host infection [44], [45]. Cyst germination was tested using zoospore suspensions from the three mutants and P6497, which formed cysts after incubation in 5% V8 liquid at 25°C. After 12 h, the percentage of abnormal cyst germination of the three mutants ranged from approximately 15 to 23%. Abnormal cyst germination affected polarized growth with highly branched and apical swelling, but there was no abnormal cyst germination in P6497, which represented a significant difference (Fig. 5A and 5B). We also observed the role of *PsVPS1* in sporangia formation, sporangia morphology, and zoospore release, and found that partial silencing of VPS1 did not affect these phenotypes.

Fungi possess cell walls made of the glucosamine polymer chitin, and algae typically possess walls made of glycoproteins and polysaccharides. However, the cell walls of oomycetes consist of β-1, 3 polysaccharides, β-1, 6 polysaccharides, cellulose, and trace amounts of chitin [46], [47]. To further explore the cause of abnormal cyst germination, we investigated the effects of cell wall on the silenced mutant. Wild-type strain 6497 and the silenced mutants were stained with Congo Red, which has a high affinity for polysaccharides. Strongly stained patches were detected along the hyphae of the silenced mutants in addition to the hyphal tips, indicative of abnormal polysaccharide accumulation. In contrast, red fluorescence was accumulated at the hyphal tips in wild-type strain (Fig. 5C). Staining with CFW was also used to probe the distribution of cellulose on the living cell wall. In the silenced mutants, patches of bright fluorescence were observed on the lateral walls of hyphae, while in the wild-type strain 6497, most of the CFW fluorescence was observed at the lateral branch and tips where cellulose was actively synthesized (Fig. S2). This was suggestive of altered cellulose distribution in the cell wall of silenced mutants. These findings indicated that the aberrant polysaccharide distribution contributed to abnormal cyst germination.

### *PsVPS1* is Required for Pathogenicity

**Figure 6:**
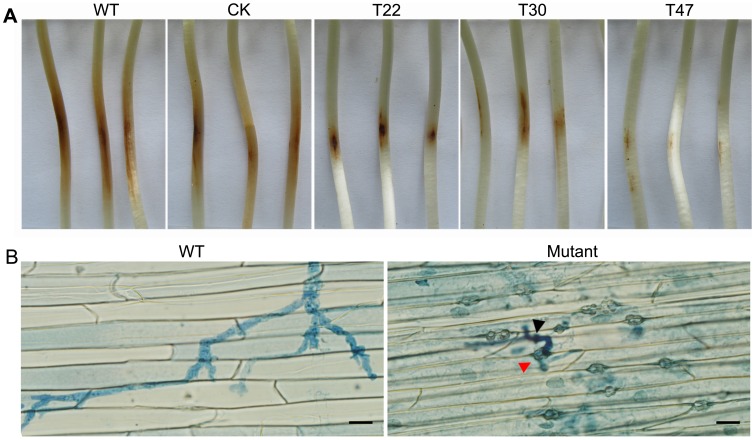
*PsVPS1* is required for pathogenicity. A. Pathogenicity tests on soybean (Hefeng 47) using zoospores from P6497 and *PsVPS1*-silenced mutants (T22, T30, and T47) inoculated on hypocotyls of etiolated seedlings. Etiolated seedlings were drop-inoculated with equal numbers of effective zoospores (100/10 µl) for 48 h, after which the samples were photographed. Experiments were repeated at least three times. **B.** Microscopic observations of invasive hyphae in soybean hypocotyls epidermal cells (infectious ostioles, red arrowhead; infectious hyphae, black arrowhead). After 48 hpi, the epidermis of seedling hypocotyls was soaked in Trypan Blue for 20 min, and then washed with SDW. The experiments were repeated three times in all mutants with similar results. Bar indicates 20 µm.

To examine the role of *PsVPS1* in soybean root, inoculation assays were performed by applying zoospore suspensions from the wild type and three silenced mutants T22, T30, and T47 on leaves and etiolated seedlings of the soybean cultivar Hefeng 47. At 2 dpi, hypocotyls of etiolated seedlings inoculated with wild-type zoospores showed typical disease symptoms and water-soaked lesions. In contrast, the three silenced mutants showed the same features with very small lesions that did not expand beyond the site of inoculation (Fig. 6A). Simultaneously, the leaves also were inoculated with zoospores and stained by Trypan Blue. Wild-type caused typical disease symptoms and the infectious hypha could extend to the whole leaf. In contrast, the silenced mutants could not produce disease symptoms with very small necrosis at the site of inoculation.

To determine whether the pathogenicity defect was associated with penetration, hypocotyls of etiolated seedlings were wounded by scratching with a pipette tip prior to inoculation with zoospore suspensions. At 2 dpi under the same conditions, disease symptoms were not expanded on the hypocotyls inoculated with the silenced mutants. However, hypocotyls of etiolated seedlings inoculated with the wild type showed typical disease symptoms (Fig. S3). We concluded that the loss of pathogenicity was not directly associated with penetration.

Hence, we examined the infectious stage and used the excised epidermal cells of hypocotyls of etiolated seedlings to examine infectious hyphae within host cells. Hypocotyls of etiolated seedlings were inoculated with an equal concentration of zoospore suspension, and at 12 hpi we observed that germinated cysts could penetrate epidermal cells in both the mutants and wild type. This demonstrated that there was no distinction between the mutants and wild type. At 48 hpi based on microscopic examinations, the wild type produced large amounts of infectious hyphae, whereas the mutants produced only small amounts of short infectious hyphae (Fig. 6B). We found that the hyphae infection was limited to the site of inoculation. We suggest that the mutants could not expand the hyphae infection. We next explored whether the reduced pathogenicity was due to the impairment of mutant growth. To eliminate the influence of the impaired growth, we observed the seedlings until 7 dpi, and disease symptoms did not spread (Fig. S4).

Plant defense responses play important roles in plant–microbe interactions. Therefore, we examined the host-derived reactive oxygen species by staining with 3, 3′-diaminobenzidine (DAB) 8 h after inoculation. The staining results showed no significant difference, indicating that the ability to remove active oxygen was not reduced in mutants. Callose deposition was also similar between the mutants and wild type. These results indicate that the pathogenic deficiency was not associated with active oxygen removal or callose deposition. To further investigate the plant defense response, the expressions of soybean defense genes EDS1, PR1, RAR1, and SGT1 were analyzed in etiolated seedling tissue by real-time PCR, which revealed no significant differences (P<0.05) between the silenced mutants and wild type (Fig. S5). These results indicated that the pathogenic deficiency was not associated with plant defense responses.

### *PsVPS1*-silenced Mutants Decrease the Activity of Extracellular Protein

**Figure 7:**
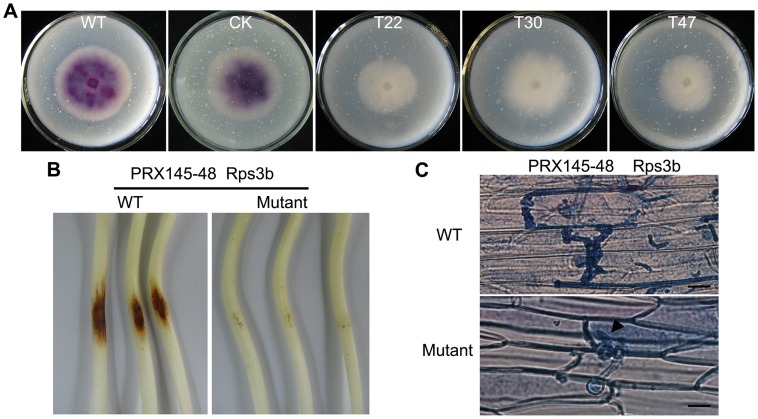
Detection of extracellular protein. **A.** Laccase activity was detected in lima bean media supplemented with 0.4 mM ABTS 4 days after inoculation. **B.** Mutants no longer induced extensive HR on PRX145-48 4-day-old seedlings. **C.** After 48 hpi, the epidermis of seedling hypocotyls was soaked in Trypan Blue for 20 min and then washed with SDW(infectious hyphae, black arrowhead). Bar indicates 20 µm.

We next examined the effect of silencing *P. sojae* vps1 on secretion of extracellular enzymes. Laccase is widely distributed in plants and fungi and is a secreted extracellular protein that can be assessed by ABTS. Laccase activity was measured in the three mutants and wild-type 6497 grown on solid lima bean medium. Significantly decreased laccase activity was observed in the three mutants (Fig. 7A). We also examined the activity of another extracellular enzyme, pectinase, but there was no significant difference between silenced mutants and wild-type 6497.

To determine whether the silenced mutants have altered secretion of effector proteins, a differential host of soybean (PRX145-48) containing the resistance protein Rps3b, were inoculated with P6497 and the silenced mutants. After 48 hpi, the silenced mutants did not induce hypersensitive response (HR) spots on PRX145-48. P6497 formed HR spots significantly (Fig. 7B). At the cellular level, infectious hyphae of P6497 or the silenced mutants were observed in PRX145-48 epidermal cells stained with Trypan Blue, but the infectious hyphae of the silenced mutants were short and did not induce hypersensitive death (Fig. 7C). Therefore, the silenced mutants impaired Rps3b-mediated resistance maybe due to decreased or defective secretion of the AVR3b protein.

## Discussion

In this study, we characterized the *P. sojae* dynamin-related protein. PsVPS1 was conserved in the sequenced oomycete genome and contained a GTP site binding domain, a middle domain, and a GTPase effector domain (GED). *PsVPS1* was required for vacuolar biogenesis, cyst germination, protein secretion, and pathogenicity.

Vacuoles play a role in the export of a variety of solutes important for eukaryotic organisms. For example, these solutes play roles in glycoprotein turnover, hydrolysis, Ca^2+^ storage, osmotic regulation, polyphosphates, pH, ion homeostasis, protein degradation, and amino acid storage. Yeast has been used to explain the mechanisms of vacuolar biogenesis, protein sorting, vacuolar transport, homotypic membrane fusion, and homeostasis [24]. More than 40 genes were identified in *S. cerevisiae* vacuolar protein sorting (VPS) and most of the *vps* mutants possessed vacuolar morphologies. Sequence analysis revealed that *P. sojae* VPS1 was a homolog of the *S. cerevisiae* vacuolar protein sorting gene vps1. The *S. cerevisiae* VPS1 mutant showed severe defects in soluble vacuolar protein sorting, such as mislocalization and secretion of newly synthesized CPY and the accumulation of aberrant membrane-enclosed structures [48]. In the filamentous fungi *A. nidulans*, disruption of VPSA resulted in poor growth and contained highly fragmented vacuoles. Our results suggested that *PsVPS1* plays a critical role in vacuolar biogenesis, with the frequent formation of fragmented vacuolar structures similar to the *S. cerevisiae* VPS1 mutant.

The vacuole is of significant interest to the membrane trafficking system, including biosynthetic and endocytic systems. Vacuoles play a direct role in long-distance nutrient transport, the regulation of hyphal extension and branching, and the induction of vital morphogenetic processes such as appressorium formation and pseudohyphal growth. In the basidiomycete *U. maydis*, fusion between two haploid sporidia of different mating types is required to form the dikaryotic hypha, which can induce tumors in meristematic tissues of the maize plant [49]. In *C. albicans*, germ tube formation involves the enlargement of the vacuole in the mother yeast cell, and most of the cytoplasm migrates into the hypha [50]. The vacuole is inherited asymmetrically during cytokinesis so that the distal compartments inherit most of the vacuole while the growing apical cell inherits most of the cytoplasm during subsequent cell cycles. Some mutants in *C. albicans* affected in vacuole inheritance and translocation have defects in hyphal development and branching frequency [51]–[55]. Disruption of VPSA in *A. nidulans* resulted in a slow-growth phenotype. Thus, the vacuole seems to play an important role in cell-cycle progression, hyphal growth, and branch initiation in fungi. Our results demonstrated that silencing of *PsVPS1* in *P. sojae* affected the colony growth rate. We simultaneously observed that cyst germination was abnormal. Microstructural examination of the silenced mutant revealed abnormal accumulation of polysaccharide after staining by Congo Red and by CFW, respectively. In Golgi body, β-Glucan synthetases have been found, and many enzymes have been found in vacuoles which may be responsible for synthesis of polysaccharide or they maybe collected there prior to delivery to the cell wall for synthesis of wall or extracellular polysaccharide [56]. This suggested that the vacuolar system could function as an alternative route for transported proteins destined for the cell surface.

In this study, pathogenicity was strongly reduced in the silenced *PsVPS1* mutants. At the macroscopic level, small lesions were observed in the silenced mutants. Seedling assays showed that at the cellular level in the epidermis of hypocotyls, the epidermis was penetrated by both the wild type and mutants at 12 hpi. After 48 hpi, the hyphal infection limited the growth of silenced mutants at specific infection sites. In fungi, some *VPS* mutants with altered vacuole function, biogenesis, and inheritance have reduced virulence. For example, in *C. albicans* the deletion of *VPS21*-mediated trafficking reduced hyphal growth and pathogenicity, *VPS28* and *VPS32* deletions resulted in defective endocytosis and pronounced defects in pathogenesis, and the *VPS34* mutant showed reduced germ tube formation and was avirulent in an infection model. Moreover, enlarged vacuoles were present in mutant cells, similar to *S. cerevisiae VPS34* [51], [57]. Since silenced *PsVPS1* mutants have impaired growth, it is possible that the reduced pathogenicity may be the result of growth defects. Seedlings were inoculated with zoospores under moist conditions and were observed for 7 days. The hyphae could not expand in the hypocotyls of etiolated seedlings, indicating that the loss of virulence was independent of the growth defect of *PsVPS1*-silenced mutants. Moreover, the expression of soybean defense genes did not differ significantly, suggesting that the pathogenic deficiency was not associated with the plant defense response. In plant and animal pathogens, it is assumed that the production of extracellular proteins occurs during all stages of pathogenic development in the host cell. It has been shown that extracellular proteins play an essential role in pathogenic development [10], [17], [58], including some enzymes and effectors of plant cell wall degradation. In *C. albicans*, deletion of the *VPS11* gene reduces secretion of Saps and lipases. *VPS34*Δ and *VPS4*Δ, which are class E vacuolar mutants, cannot secrete SAP2 protein [59]. *VPS1*Δ has reduced extracellular proteinase activity and lipolytic activity under repressing conditions [21]. The *PsVPS1*-silenced mutants secreted less extracellular laccase when grown on ABTS lima bean agar, but pectinase did not exhibit reduced activity. Thus, silenced *PsVPS1* did not completely block enzyme secretion. The compatible interactions demonstrated that *PsVPS1* is critical during infection on Hefeng 47, but in incompatible interactions we found that silenced mutants could not induce HR on PRX145-48. In *M. grisea*, when APT2 and LHS1 (which affect extracellular protein secretion) were knocked out, these mutants lost the ability to elicit the hypersensitive reaction on rice cultivars resistant to *M. grisea* Guy11. Further studies demonstrated that mutants affect AVR-Pita function and secretion [17], [58]. Overall, it is possible that the pathogenic deficiency might be due to reduced activity of extracellular proteins.

Thus, we have demonstrated that *P. sojae* VPS1 is involved in cyst germination, extracellular protein secretion, and pathogenicity, suggesting that it plays an important role in the pre-vacuolar secretory pathway. Secreted effector proteins are important for the successful invasion of plant cells and it is possible that some of these proteins play important roles in pathogenesis. Thus, characterization of these genes may lead to novel strategies to control soybean root rot disease. Further experiments are required to determine whether silencing of *PsVPS1* affects the secretion of effectors and why infected mycelia in plant cells cannot expand.

## Supporting Information

Figure S1 **Phylogenetic dendrograms of PsVPS1 protein sequences from different organisms.** The phylogenetic tree of PsVPS1 was generated using the MEGA 4 program, with neighbor joining, 1000 bootstraps and amino acid P-distance, based on alignment of the full sequences of VPS1 families. The sequences of VPS1 families were obtained from the following organisms: *Phaeodactylum tricornutum* (XP_002181636), *Mus musculus* (NP_690029), *Magnaporthe oryzae* (XP_364672), *Saccharomyces cerevisiae* (NP_012926), *Phytophthora sojae* (109490), *Phytophthora ramorum* (72119), *Phytophthora capsici* (576775), *Homo sapiens* (NP_036193), *Phytophthora infestans* (XP_002908808), *Thalassiosira pseudonana* (XP_002296064), *Arabidopsis thaliana* (NP_567931), and *Oryza sativa* (EEC72053).
(TIF)

Figure S2 **Silencing of** ***PsVPS1*** **altered the distribution of cellulose.** In the wild-type strain P6497, CFW fluorescence was mainly distributed at the hyphae and branches, whereas in the transformant fluorescence was not restricted to growing apices and was also observed on the lateral walls along the hyphal axe. Bar indicates 20 µm.
(TIF)

Figure S3 **Penetration test on soybean (Hefeng 47) using zoospores from P6497 and** ***PsVPS1*** **-silenced mutants (T22, T30 and T47) to hypocotyls of seedlings.** Wounded seedlings were drop-inoculated with equal numbers of effective zoospores (100/10 µl) for 48 h, after which the samples were photographed. The experiments were repeated at least three times.
(TIF)

Figure S4 **The growth ratio does not affect pathogenicity.** Seedlings of 4-day-old soybean (Hefeng 47), growing without light, were drop-inoculated with equal numbers of effective zoospores (100/10 µl) for 7 d, after which the samples were photographed.
(TIF)

Figure S5 **Expression of plant defense-related genes.** Bar chart showing the expression of soybean EDS1 (enhanced disease susceptibility 1), SGT1 (suppressor of the G2 allele of skp1), PR1 (pathogenesis related gene 1) and RAR1 (required for Mla12 resistance) 12 hpi by wild type 6497, the control transformant (CK) Psvps1-silenced mutants (T22, T30 and T47), respectively. The experiments were repeated at least three times.
(TIF)
