# Supplementary figures and images for: PsVPS1, a Dynamin-Related Protein, Is Involved in Cyst Germination and Soybean Infection of Phytophthora sojae
Source: PLoS One. 2013 Mar 14;8(3):e58623. doi: 10.1371/journal.pone.0058623 (PMC3597732; doi:10.1371/journal.pone.0058623)

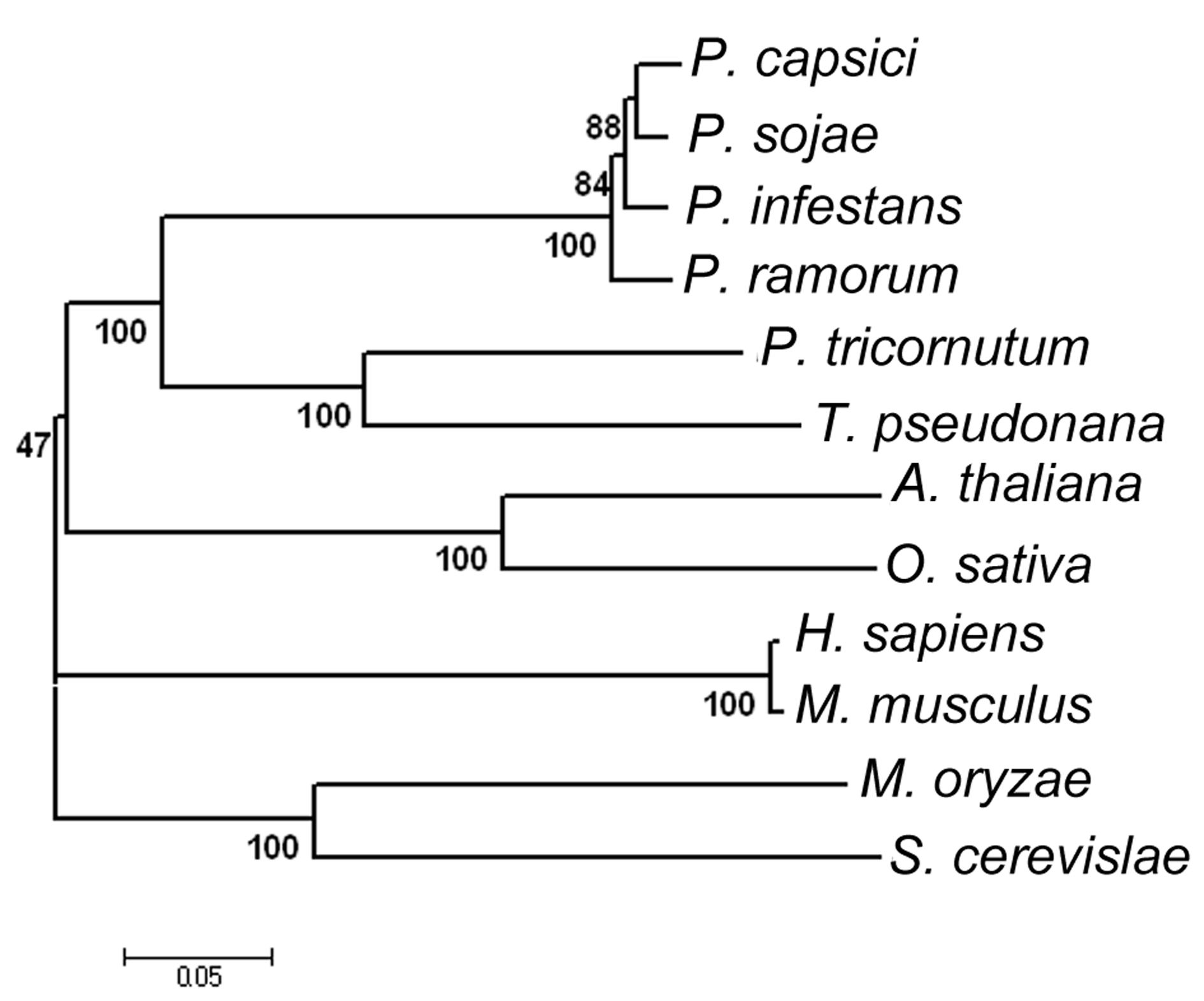

Supplement: Figure S1 — Phylogenetic dendrograms of PsVPS1 protein sequences from different organisms. The phylogenetic tree of PsVPS1 was generated using the MEGA 4 program, with neighbor joining, 1000 bootstraps and amino acid P-distance, based on alignment of the full sequences of VPS1 families. The sequences of VPS1 families were obtained from the following organisms: Phaeodactylum tricornutum (XP_002181636), Mus musculus (NP_690029), Magnaporthe oryzae (XP_364672), Saccharomyces cerevisiae (NP_012926), Phytophthora sojae (109490), Phytophthora ramorum (72119), Phytophthora capsici (576775), Homo sapiens (NP_036193), Phytophthora infestans (XP_002908808), Thalassiosira pseudonana (XP_002296064), Arabidopsis thaliana (NP_567931), and Oryza sativa (EEC72053). (TIF) [file pone.0058623.s001.tif]

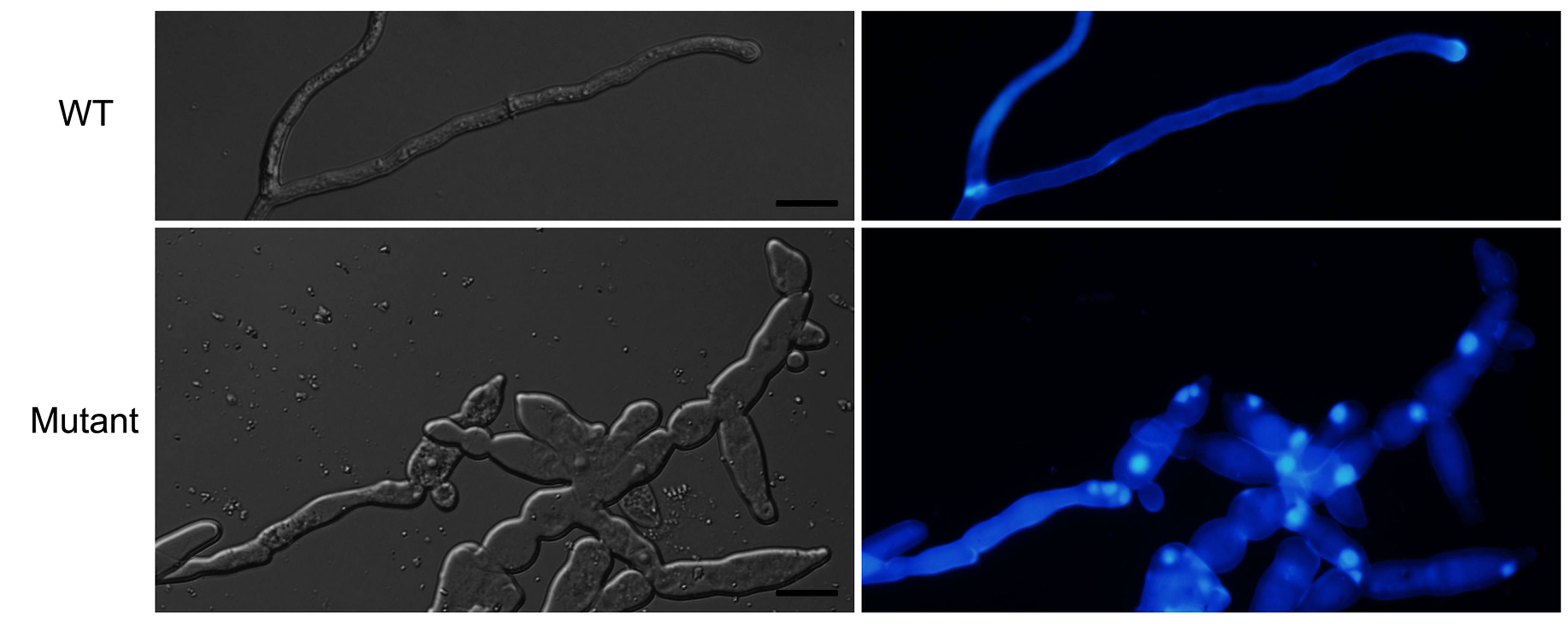

Supplement: Figure S2 — Silencing of PsVPS1 altered the distribution of cellulose. In the wild-type strain P6497, CFW fluorescence was mainly distributed at the hyphae and branches, whereas in the transformant fluorescence was not restricted to growing apices and was also observed on the lateral walls along the hyphal axe. Bar indicates 20 µm. (TIF) [file pone.0058623.s002.tif]

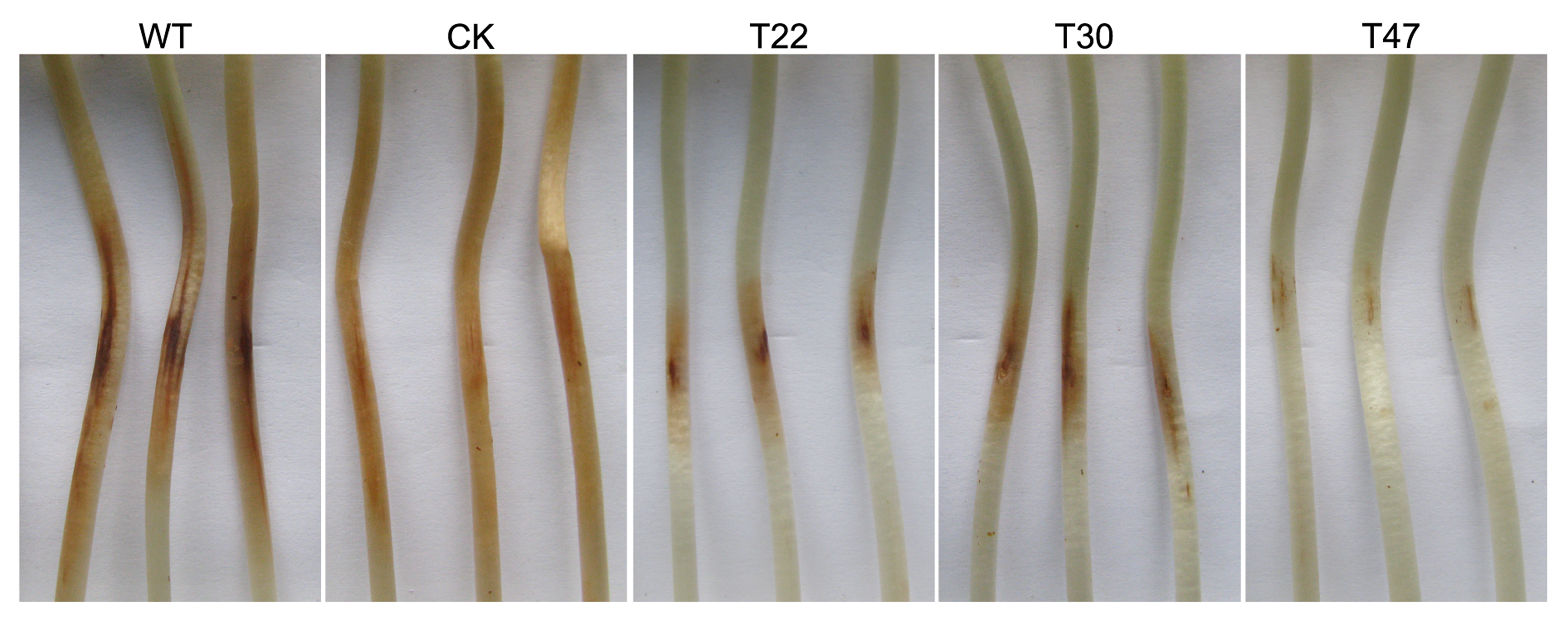

Supplement: Figure S3 — Penetration test on soybean (Hefeng 47) using zoospores from P6497 and PsVPS1 -silenced mutants (T22, T30 and T47) to hypocotyls of seedlings. Wounded seedlings were drop-inoculated with equal numbers of effective zoospores (100/10 µl) for 48 h, after which the samples were photographed. The experiments were repeated at least three times. (TIF) [file pone.0058623.s003.tif]

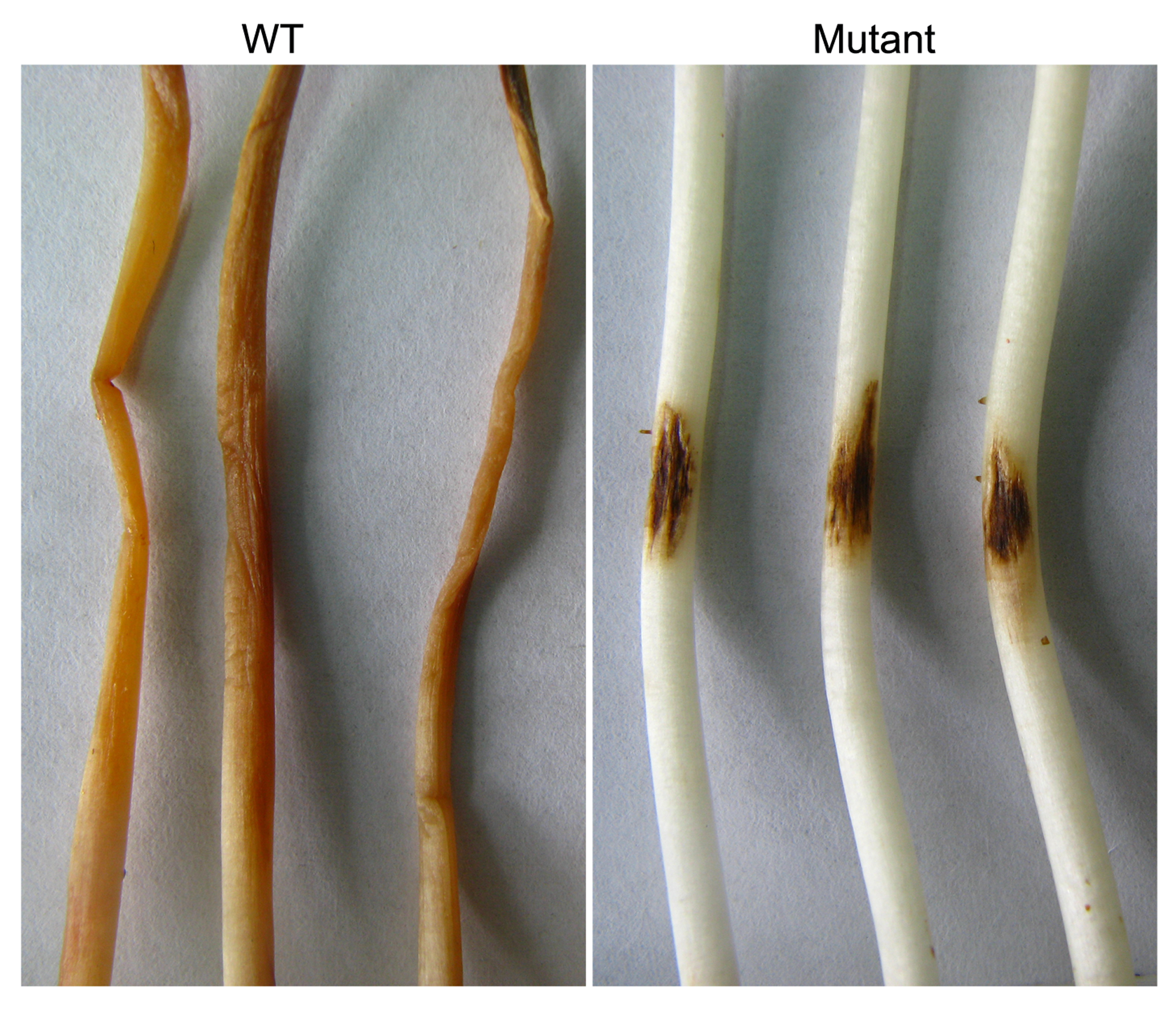

Supplement: Figure S4 — The growth ratio does not affect pathogenicity. Seedlings of 4-day-old soybean (Hefeng 47), growing without light, were drop-inoculated with equal numbers of effective zoospores (100/10 µl) for 7 d, after which the samples were photographed. (TIF) [file pone.0058623.s004.tif]

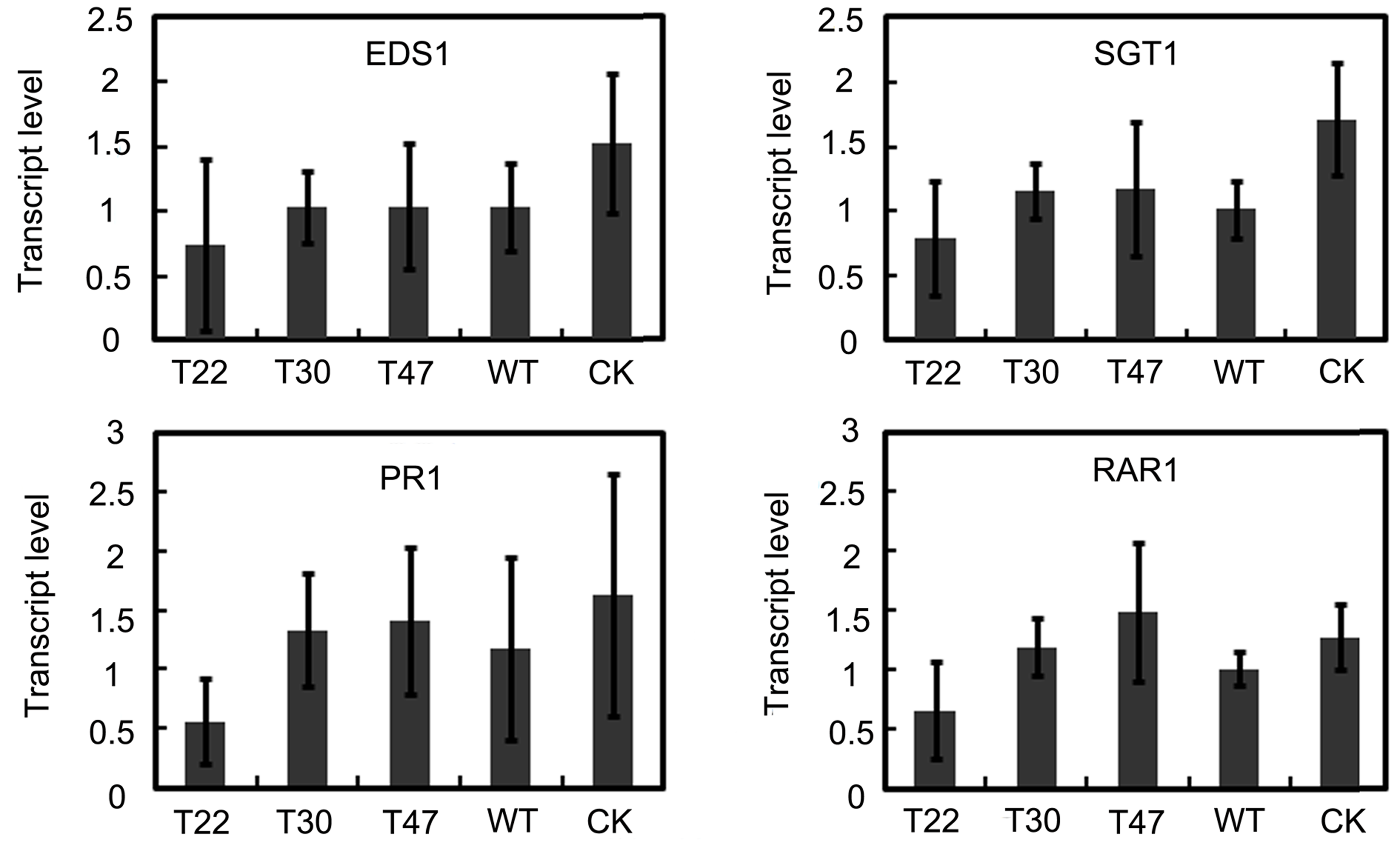

Supplement: Figure S5 — Expression of plant defense-related genes. Bar chart showing the expression of soybean EDS1 (enhanced disease susceptibility 1), SGT1 (suppressor of the G2 allele of skp1), PR1 (pathogenesis related gene 1) and RAR1 (required for Mla12 resistance) 12 hpi by wild type 6497, the control transformant (CK) Psvps1-silenced mutants (T22, T30 and T47), respectively. The experiments were repeated at least three times. (TIF) [file pone.0058623.s005.tif]
